# Noninvasive Risk Stratification Based on Renal Tubular Injury Phenotypes: A Deep Learning Study for Predicting Vesicoureteral Reflux in Children

**DOI:** 10.1111/jcmm.71172

**Published:** 2026-05-10

**Authors:** Hongzhou Lin, Zeyu Cui, Zhantian Zhang, Yifan Luo, Jianwei Shuai, Kai Zhao, Yunlong Zhou, Dexuan Wang

**Affiliations:** ^1^ Department of Pediatrics The Second Affiliated Hospital and Yuying Children's Hospital of Wenzhou Medical University Wenzhou China; ^2^ Wenzhou Medical University Wenzhou China; ^3^ Joint Medical Engineering Interdisciplinary Research Center of Wenzhou Institute UCAS The Second Affiliated Hospital of Wenzhou Medical University Wenzhou China; ^4^ Wenzhou Institute University of Chinese Academy of Sciences Wenzhou China

**Keywords:** cortical uptake phenotype, inflammation and fibrosis, oxidative stress, static renal 99mTc‐DMSA scintigraphy, tubulointerstitial injury, urinary tract infection, vesicoureteral reflux

## Abstract

Vesicoureteral reflux (VUR) can cause retrograde urine flow under voiding pressure, facilitating ascending bacterial colonisation and recurrent inflammatory responses. These processes trigger a cascade of cellular and molecular events—innate immune activation, pro‐inflammatory cytokine release, oxidative stress, apoptosis and extracellular matrix deposition—thereby promoting tubulointerstitial remodelling and increasing the risk of renal parenchymal injury and scarring. Static renal 99mTc‐DMSA scintigraphy primarily reflects tracer uptake by proximal tubular cells in the renal cortex and can serve as an integrated phenotypic readout of tubular dysfunction and focal cortical involvement. However, its clinical interpretation remains experience‐dependent and lacks reproducible quantitative criteria, while voiding cystourethrography (VCUG), the diagnostic and grading gold standard for VUR, is limited by invasiveness and procedural burden. In this study, we collected DMSA data from 346 children with febrile urinary tract infection treated at the Second Affiliated Hospital of Wenzhou Medical University between January 2019 and January 2023 and developed a deep learning model (MedSwinNet) for VUR risk stratification. Built on a Swin Transformer backbone and enhanced with multi‐scale representation fusion, a convolutional block attention module and a gated selection strategy, MedSwinNet was designed to sensitively capture phenotypic signals such as reduced proximal tubular uptake and focal cortical defects while improving robustness. On the test set, the model achieved accuracies of 0.8290 under the severe‐side input setting and 0.7997 under the bilateral‐side input setting, demonstrating stable discriminative performance and favourable generalisation. Quality control analyses indicated broadly consistent distributions of key image quality metrics across data splits, mitigating potential bias from dataset shift. Collectively, deep learning–based decoding of tubular dysfunction–related phenotypic readouts enables noninvasive quantification of VUR‐associated renal involvement, supports decision‐making on whether VCUG is warranted and may reduce unnecessary invasive procedures while improving clinical risk‐stratified management.

**Abbreviations**
AUC: area under the receiver operating characteristic curve
CBAM: convolutional block attention module
DCA: decision curve analysis
DMSA: dimercaptosuccinic acid
FN: false negative
FP: false positive
Grad‐CAM: gradient‐weighted class activation mapping
IQR: interquartile range
KS: Kolmogorov–Smirnov test
MaxViT: multi‐axis vision transformer
MCU: micturating cystourethrography
MLP: multilayer perceptron
MWU: Mann–Whitney *U* test
PACS: picture archiving and communication system
QC: quality control
ResNet: residual network
ROC: receiver operating characteristic (curve)
ROI: region of interest
SW‐MSA: shifted‐window multi‐head self‐attention
TN: true negative
TP: true positive
TRIPOD+AI: Transparent Reporting of a multivariable prediction model for Individual Prognosis or Diagnosis + Artificial Intelligence
UTI: urinary tract infection
VCUG: voiding cystourethrography
VUR: vesicoureteral reflux
W‐MSA: window‐based multi‐head self‐attention

## 1 Introduction

Urinary tract infection (UTI) is a common infectious disease in children, typically caused by bacteria, viruses or fungi and may involve the kidneys, ureters, bladder and urethra. Depending on the anatomical site, UTIs are generally classified as upper urinary tract infections (primarily affecting the kidneys and ureters) or lower urinary tract infections (primarily affecting the bladder and urethra). Among these, upper urinary tract infection—particularly pyelonephritis—is of greater clinical concern because it may lead to impaired renal function and toxin accumulation.

Because of anatomical immaturity and an underdeveloped immune system, children with upper urinary tract infection often present with nonspecific symptoms (e.g., fever, reduced appetite, vomiting, irritability), which can be overlooked or misdiagnosed. Of particular importance is vesicoureteral reflux (VUR), a pathological condition in which urine refluxes from the bladder into the ureters and kidneys under voiding pressure. VUR can facilitate ascending infection and renal scarring and represents a major contributor to recurrent UTI and long‐term renal injury in children [1]. Notably, VUR resolves spontaneously in a proportion of children during growth and development and the likelihood of resolution is associated with reflux grade and ureteral anatomical parameters (e.g., ureteral diameter ratio) [2].

Epidemiological data indicate that approximately 30% of children with a first febrile UTI have VUR as an underlying structural abnormality, whereas the detection rate in otherwise healthy children is approximately 2% [3]. Clinically, VUR is commonly graded from I to V according to the extent of reflux and associated anatomic dilation: grade I is confined to the ureter; grade II reaches the renal pelvis without significant dilation; grades III–V show progressively greater dilation and distortion of the ureter, renal pelvis and calyces, and severe cases may exhibit marked ureteral tortuosity and increasing renal parenchymal involvement. Overall, low‐grade VUR is more likely to resolve during childhood, whereas high‐grade or bilateral VUR tends to persist and is associated with a higher risk of renal injury and scarring. Therefore, timely detection and accurate assessment of VUR severity are critical components of paediatric UTI management.

Voiding cystourethrography (VCUG/MCU) is currently regarded as the gold standard for diagnosing and grading VUR. During VCUG, contrast material is instilled via catheterisation and X‐ray images acquired during voiding can directly demonstrate reflux and support grade assignment [3]. Despite its diagnostic accuracy, paediatric use of VCUG is limited by procedural invasiveness and radiation exposure. Under modern low‐dose protocols, the median effective whole‐body dose for a single paediatric VCUG is approximately 0.04 mSv (range, 0.01–1.0 mSv) [4] and a mean effective dose of approximately 0.20 mSv has also been reported by some centres [5]. In addition, catheterisation may cause discomfort and confer a small risk of urinary tract infection. In clinical practice, management strategies for VUR typically integrate reflux grade, risk of recurrent infection and the extent of renal parenchymal involvement, and may include surveillance, prophylactic antibiotics, endoscopic injection or surgical intervention [6]. Accordingly, there remains a need to optimise risk stratification and refine indications for invasive testing while reducing unnecessary procedural burden.

**FIGURE 1:**
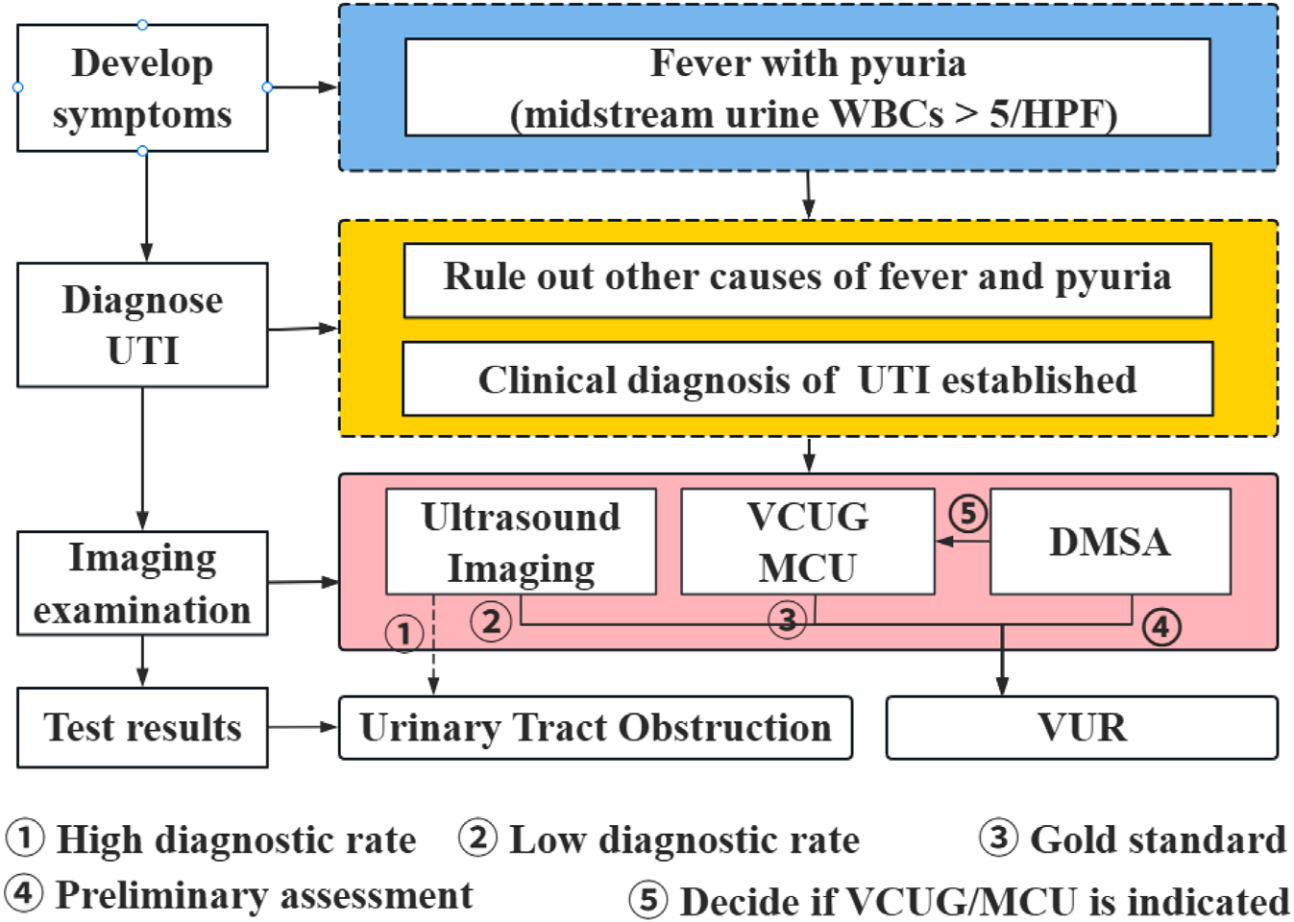
**Clinical workflow from febrile presentation to VUR evaluation in children.** After symptom onset, if the child presents with fever accompanied by pyuria/leukocyturia (midstream urine WBC > 5/HPF), other causes of fever and pyuria are first excluded and a clinical diagnosis of UTI is established. Imaging evaluation then follows: Ultrasound is used as the first‐line screening modality to detect structural renal abnormalities and urinary tract obstruction, but has limited capability for identifying VUR (①–②). Static renal DMSA scintigraphy is used for an initial assessment of cortical involvement/scarring (④), based on which the indication for further VCUG/MCU is determined (⑤). VCUG/MCU remains the gold standard for definitive diagnosis and grading of VUR (③). DMSA, static renal 99mTc‐dimercaptosuccinic acid scintigraphy; HPF, high‐power field; MCU, micturating cystourethrography; UTI, urinary tract infection; VCUG, voiding cystourethrography; VUR, vesicoureteral reflux; WBC, white blood cell.

In routine care, children often present with unexplained fever and are suspected to have UTI, after which they enter an imaging‐based evaluation and stratification pathway (Figure 1). Ultrasound is typically used as a first‐line screening tool to identify structural abnormalities such as hydronephrosis and ureteral dilation. For patients with positive screening findings or recurrent UTI, further evaluation focuses on whether renal parenchymal involvement suggestive of VUR is present, thereby informing whether VCUG is warranted for definitive diagnosis and grading (Figure 1).

Static renal dimercaptosuccinic acid (DMSA) scintigraphy has been increasingly adopted as a relatively gentle, low‐radiation nuclear medicine approach for assessing VUR‐related renal parenchymal injury and for providing a functional phenotypic readout. Following administration of radiolabeled DMSA, tracer uptake by proximal tubular cells can be visualised with gamma‐camera imaging, reflecting cortical uptake patterns and renal functional status. Under normal conditions, DMSA scintigraphy shows a well‐defined renal contour with homogeneous tracer distribution, whereas renal injury may manifest as focal photopenic defects or morphological alterations [7]. Although DMSA scintigraphy does not directly depict the reflux pathway, it provides important information for evaluating renal injury and can assist in determining whether additional VCUG should be performed [3]. Prior work also suggests that DMSA is valuable for identifying VUR‐associated renal scarring or injury and can serve as an important basis for downstream testing decisions and follow‐up assessment [8]. Moreover, the effective dose associated with DMSA scintigraphy is generally within acceptable limits for paediatric diagnostic examinations; however, comparisons with VCUG depend on device parameters and protocol settings and should be interpreted in the context of specific study conditions [9, 10]. Nevertheless, DMSA scintigraphy has a non‐negligible false‐positive rate and its clinical interpretation remains experience‐dependent and lacks reproducible quantitative criteria, limiting broader adoption for early VUR screening and risk stratification.

In recent years, artificial intelligence (AI), particularly deep learning, has advanced rapidly in biomedical data analysis, enabling automated feature extraction and pattern recognition to improve efficiency and consistency in disease identification and risk assessment. AI has shown strong potential across medical tasks by supporting clinicians in optimising diagnostic decisions, reducing errors and facilitating individualised management [11]. In representation learning for medical applications, Swin Transformer has demonstrated favourable performance due to its hierarchical architecture and capacity for multi‐scale modelling. Representative studies include Swin UNETR with self‐supervised pretraining for 3D tasks [12], HRSTNet integrating Swin modules with HRNet to preserve high‐resolution features [13], FE‐SwinUper combining feature‐enhanced Swin Transformer with UPerNet using attention and adaptive fusion mechanisms [14] and mST‐MLAFF leveraging local–global attention with multi‐level adaptive fusion for high‐performance segmentation and classification [15]. These developments provide methodological support for adopting a Swin Transformer backbone in our framework.

For VUR specifically, existing AI studies have largely focused on automated recognition and grading based on VCUG. Ergün et al. developed a CNN‐based grading approach with transfer learning and data augmentation and reported strong performance on small datasets [16]. Li et al. proposed Deep‐VCUG and validated it on multicentre data, improving robustness through ensemble strategies and enhancing inter‐reader agreement across clinicians with different experience levels [17]. Eroglu et al. employed hybrid feature extraction and feature selection strategies to improve diagnostic robustness [18]. Subsequent work further incorporated multi‐head convolution and attention mechanisms to improve grading performance on multi‐institutional data [19], and traditional machine learning approaches combined with radiomics features have also been explored for quantitative prediction of reflux severity with encouraging discriminative performance [20]. Collectively, these studies indicate that AI can improve efficiency and objectivity in cystourethrography interpretation; however, VCUG is inherently invasive and primarily reflects reflux pathways and grades, rather than directly capturing renal functional impairment, which may limit its utility for longitudinal follow‐up and comprehensive risk assessment.

In contrast, DMSA provides a functional phenotypic readout of renal parenchymal involvement that aligns more closely with the clinical need for ‘renal injury risk stratification–guided testing decisions.’ Therefore, we developed and validated MedSwinNet, a Swin Transformer–based deep learning model for predicting VUR using DMSA data. By incorporating multi‐scale feature fusion, a convolutional block attention module (CBAM) [21], and a gated strategy, our approach aims to improve predictive performance and provide reproducible decision support regarding whether VCUG should be performed, thereby reducing unnecessary invasive procedures and radiation‐related burden while promoting AI‐enabled risk stratification and translational evaluation in paediatric urologic disease.

## 2 Materials and Methods

**FIGURE 2:**
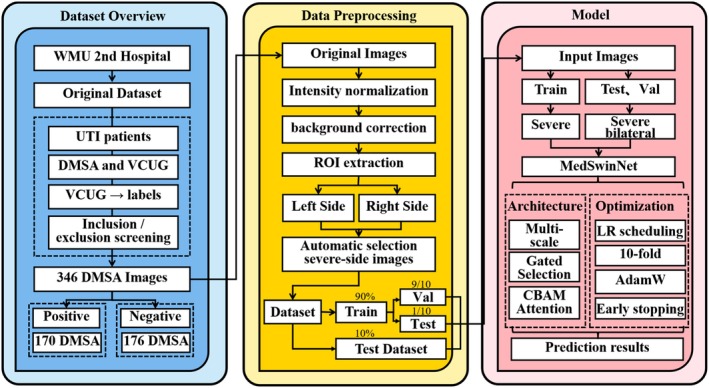
**Overall framework for DMSA‐based VUR prediction: Dataset, preprocessing and the MedSwinNet model.** The left panel summarises the dataset: Imaging data from febrile UTI patients at the Second Affiliated Hospital of Wenzhou Medical University (WMU) were screened using predefined inclusion/exclusion criteria, yielding 346 DMSA scans. VUR labels were generated using VCUG as the gold standard (170 VUR‐positive and 176 VUR‐negative cases). The middle panel illustrates the preprocessing pipeline: After intensity normalisation and background correction, the renal region of interest (ROI) was extracted and the left and right kidneys were separated; for the severe‐side setting, one kidney was then selected programmatically according to the predefined side‐selection rule. The dataset was then split, with 90% of samples used as a development cohort and the remaining 10% reserved as an independent test set; the development cohort was subsequently used for 10‐fold cross‐validation. The right panel shows the model and training settings: Only severe‐side inputs were used during training, whereas both severe‐side and bilateral‐side inputs were evaluated during validation and testing to assess generalisation across input configurations. MedSwinNet integrates multi‐scale feature fusion, gated selection and a CBAM attention module, and is trained/evaluated using the AdamW optimiser, learning‐rate scheduling, early stopping and 10‐fold evaluation to produce final predictions and performance metrics.

As illustrated in the study workflow (Figure 2), the overall pipeline comprised three stages. First, febrile UTI patients who underwent both DMSA scintigraphy and VCUG at the Second Affiliated Hospital of Wenzhou Medical University were screened according to predefined inclusion and exclusion criteria. A total of 346 DMSA scans were collected and labelled as VUR‐positive or VUR‐negative based on VCUG findings. Second, the raw DMSA scans were subjected to intensity normalisation, background correction, renal region‐of‐interest (ROI) extraction and left–right kidney segmentation [22], after which one kidney was selected under the severe‐side setting according to the predefined side‐selection rule and used as model input. Third, the severe‐side input was fed into the proposed MedSwinNet model for representation learning via multi‐scale feature fusion, gated feature selection and CBAM‐based attention. Model training was performed using 10‐fold cross‐validation with the AdamW optimiser, learning‐rate scheduling and early stopping, and the model outputs VUR predictions under the severe‐side and bilateral‐side settings.

### 2.1 Dataset and Eligibility Criteria

This was a single‐centre retrospective study. We screened paediatric inpatients with febrile urinary tract infection (UTI) who were admitted to the Department of Paediatric Nephrology, the Second Affiliated Hospital of Wenzhou Medical University, between January 2019 and January 2023. All enrolled patients met the domestic diagnostic criteria described in the Guideline for the Diagnosis and Management of Paediatric Urinary Tract Infection (2021 Edition) and had complete clinical records.

Importantly, the cohort was not limited to children with a first febrile UTI. Instead, it included both first‐episode and recurrent febrile UTI cases. In this retrospective study, first‐episode UTI was defined as the absence of a documented prior history of UTI in the medical record, whereas recurrent UTI was defined based on a documented prior history of UTI episodes or chart descriptions such as ‘recurrent infection’ or ‘recurrent urinary tract infection.’

All included children underwent both static renal 99mTc‐DMSA scintigraphy and voiding cystourethrography (VCUG) during the same hospitalisation, and the corresponding imaging data and report results were collected for subsequent analysis. In addition to imaging data, we collected relevant clinical variables at the time of DMSA/VCUG evaluation, including sex, age, renal function indices, presence and degree of hydronephrosis, history of recurrent UTI and renal ultrasonography findings.

VUR status was determined using VCUG as the reference standard, according to the diagnostic criteria in the Expert Consensus on Primary Vesicoureteral Reflux in Children (2019 Edition). Based on VCUG findings, the dataset comprised 170 VUR‐positive cases and 176 VUR‐negative cases.

Patients were excluded if they had:

1. severe bilateral obstructive renal disease;
2. renal insufficiency;
3. obvious renal morphological or positional abnormalities on ultrasonography; or
4. other severe bacterial infectious diseases.

### 2.2 Preprocessing

#### 2.2.1 DMSA Acquisition and Standardisation

Static renal DMSA scintigraphy was performed using the same imaging system under standardised operating procedures. Scans were acquired 1 h after radiotracer injection, with consistent acquisition/display settings (e.g., window width and window level). All images were archived and managed centrally in the PACS system.

#### 2.2.2 Background Correction

To reduce errors introduced by physiological uptake in adjacent tissues, background correction was performed using integral background subtraction. Background ROIs were selected in nearby low‐uptake regions (upper‐left to the left kidney and upper‐right to the right kidney). Otsu's thresholding was applied to automatically segment the kidney ROI and background ROI, after which grayscale values were computed and corrected accordingly.

#### 2.2.3 Cropping and Resizing

Based on the background‐corrected images, Otsu's method was used to delineate bilateral kidney contours. A midline of the two kidneys was computed and kept fixed, and symmetric processing was applied to obtain a complete bilateral renal outline. The bilateral kidney region was then cropped using the minimum bounding rectangle enclosing both kidneys, with at least a 10‐pixel margin retained on each side (top/bottom/left/right). Cropped images were adjusted to an aspect ratio of 2:1 and uniformly resized to 224 × 448 pixels to ensure consistent input dimensions.

#### 2.2.4 Severe‐Side Kidney Selection

For the severe‐side setting, one kidney was selected from the bilateral DMSA images for each patient as the model input. When bilateral VUR grades were different, the kidney with the higher VUR grade was selected. When bilateral VUR grades were identical, the kidney with the lower mean tracer uptake (lower mean intensity) was selected. For VUR‐negative cases, where no reflux was identified on either side, one side was randomly selected using a fixed random seed to ensure reproducibility. This side‐selection procedure was implemented programmatically and applied consistently across the dataset.

Because the severe‐side strategy used the reference‐standard VUR grading for side selection when bilateral grades differed, it should be regarded as a label‐informed, lesion‐focused research setting intended to enhance learning of the more severely affected renal phenotype. To provide a complementary and more deployment‐relevant assessment, we also evaluated model performance under the bilateral‐side setting.

#### 2.2.5 Data Augmentation and Normalisation

Random horizontal flipping, random rotation (±15°), random cropping and brightness/contrast perturbations were applied for data augmentation to improve model robustness. All images were normalised and converted into tensors as model inputs. A fixed random seed was used to ensure experimental reproducibility.

### 2.3 MedSwinNet Model Design

**FIGURE 3:**
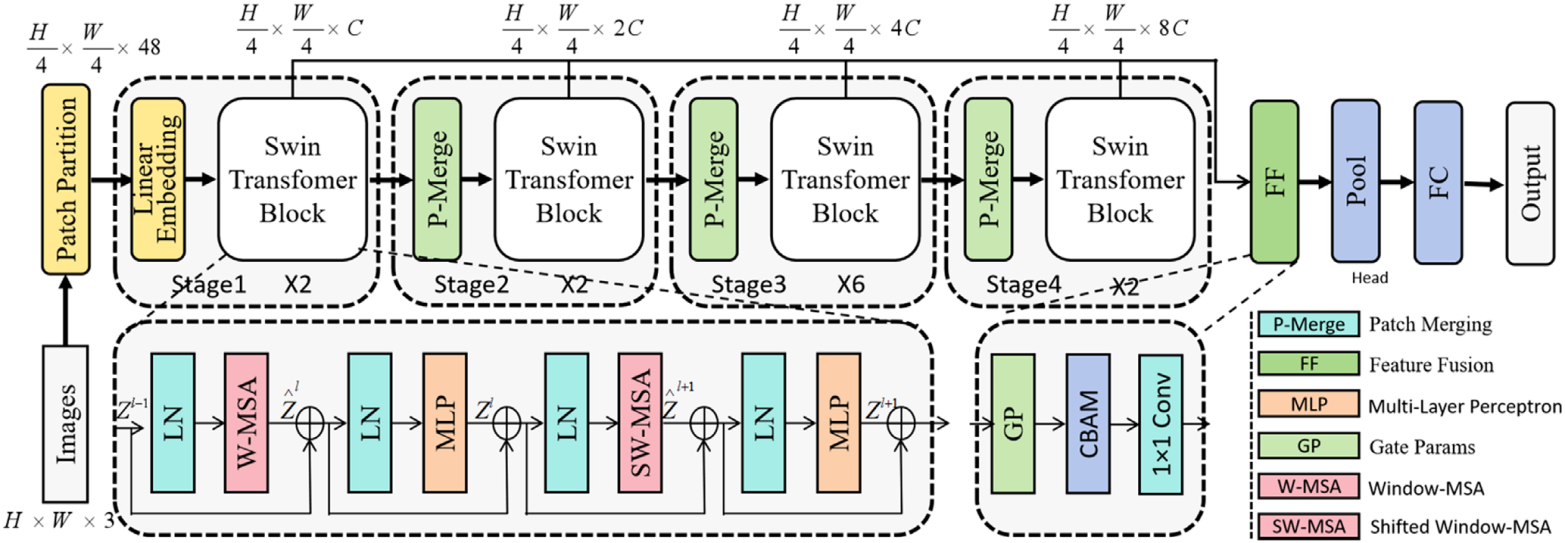
**Schematic overview of the MedSwinNet architecture.** The model takes a DMSA renal ROI as input. Initial feature representations are obtained via patch partitioning and linear embedding, after which multi‐scale features are progressively extracted through four hierarchical Swin Transformer stages. Between stages, patch merging (P‐Merge) performs spatial downsampling and channel expansion. Within each stage, layer normalisation (LN), window‐based multi‐head self‐attention (W‐MSA/SW‐MSA) and a feed‐forward network (MLP/FF) are used to jointly model local texture patterns and long‐range dependencies. To enhance lesion‐relevant representations, a gating‐based selection module (Gate, GP) is applied to high‐level features for adaptive cross‐level fusion, followed by feature recalibration using CBAM attention and a 1 × 1 convolution. Finally, global pooling (Pool) and a fully connected (FC) layer produce the VUR classification output.

DMSA scintigraphy is widely used as an important tool for assessing renal injury and has been applied in clinical evaluation of VUR. However, DMSA scans often exhibit intensity inhomogeneity, blurred boundaries and structural degradation. In particular, when lesions have not markedly progressed or renal function is only mildly impaired, conventional feature extraction may fail to capture subtle changes, posing substantial challenges for deep learning–based VUR prediction. To address these issues, we built upon the Swin Transformer architecture and introduced three key modifications to improve the model's sensitivity to subtle abnormalities in DMSA scans and ultimately enhance VUR prediction performance. The architecture of the proposed MedSwinNet model is shown in Figure 3.

#### 2.3.1 Multi‐Scale Feature Extraction and Unified Fusion

The Swin Transformer backbone provides strong hierarchical representation learning by extracting semantic information at multiple scales across four stages. In the original design, however, shallow features are typically used only as intermediate representations and do not directly contribute to the final decision. This limitation is particularly relevant for detecting subtle abnormalities in medical data (e.g., focal reductions in renal cortical uptake or early scarring). To enhance sensitivity to weak signals, we extracted intermediate features after each stage and applied bilinear interpolation to uniformly downsample them to the spatial resolution of Stage 4, enabling feature fusion at a common scale.

Let the feature map at each stage be defined as follows:

(1) $$F_{i}\in {\mathbb{R}}^{C_{i}\times H_{i}\times W_{i}},i=1,2,3,4$$

To unify the spatial scale, the feature maps from the first three stages were downsampled to the resolution of Stage 4:

(2) $${\tilde{F}}_{i}=\text{Down}\left(F_{i}\right),{\tilde{F}}_{i}\in {\mathbb{R}}^{C_{i}\times H_{4}\times W_{4}},i=1,2,3$$

This design allows the model to preserve fine‐grained details from shallow layers while retaining high‐level semantics from deeper layers, thereby improving its ability to recognise complex lesions and enhancing the flexibility and representational capacity of multi‐scale feature fusion.

#### 2.3.2 Gated Feature Selection and Optimisation

In medical data, redundant information may interfere with the extraction of discriminative cues. To mitigate this issue, we introduced a gated mechanism to automatically select the most informative features. By adaptively reweighting feature channels, the gating module optimises the model's attention to different features, thereby improving discriminative ability and robustness.

Specifically, during feature fusion, the gating mechanism uses learnable parameters to adjust the contribution of different feature channels, enabling the model to flexibly emphasise the most discriminative information under varying conditions. This design improves adaptability in complex medical settings and helps the model more effectively identify and process salient features.

Accordingly, we introduced a learnable gating mechanism in the fusion process to assign different weights to features from each stage:

(3) $$\alpha_{i}=\frac{\exp\left(g_{i}\right)}{\sum_{j=1}^{4}\exp\left(g_{j}\right)},\sum_{i=1}^{4}\alpha_{i}=1$$

where $g_{i}\in \mathbb{R}$ is a learnable parameter.

The final fused feature representation is given by:

(4) $$F_{\text{fused}}=\sum_{i=1}^{4}\alpha_{i}\cdot {\tilde{F}}_{i}$$

Building on the Swin Transformer backbone, this study proposes an efficient and highly interpretable model for VUR prediction by incorporating three key innovations: multi‐scale feature extraction and fusion, CBAM‐based attention enhancement and gated feature selection. The proposed model is designed to sensitively capture subtle abnormalities in DMSA scans and to provide an intelligent decision‐support tool that may help reduce unnecessary VCUG examinations and thereby lower radiation‐related burden in children. Collectively, these innovations improve the model's capability to process DMSA data while enhancing robustness and practical utility.

#### 2.3.3 CBAM‐Based Attention Enhancement

The complex background in DMSA scans can interfere with the identification of clinically relevant regions, particularly when renal structures are poorly defined or intensity distribution is inhomogeneous. In such cases, redundant background information may degrade discriminative performance. To address this issue, we introduced the convolutional block attention module (CBAM) after feature fusion to further strengthen the model's focus on salient regions.

The channel‐attention computation is defined as follows:

(5) $$M_{c}\left(F\right)=\sigma \left(\mathrm{MLP}\left(\text{AvgPool}\left(F\right)\right)+\mathrm{MLP}\left(\text{MaxPool}\left(F\right)\right)\right)$$

Subsequently, the channel attention map is element‐wise multiplied with the input feature to obtain the channel‐enhanced feature:

(6) $$F^{\prime }=M_{c}\left(F\right)⊙F$$

Based on this, the spatial attention map is further calculated:

(7) $$M_{s}\left(F^{\prime }\right)=\sigma \left(f^{7\times 7}\left[{\text{AvgPool}}_{\mathrm{ch}}\left(F^{\prime }\right);{\text{MaxPool}}_{\mathrm{ch}}\left(F^{\prime }\right)\right]\right)$$

The final CBAM output is computed as follows:

(8) $$F_{\text{cbam}}=M_{s}\left(F^{\prime }\right)⊙F^{\prime }$$

where $\sigma \left(\cdot \right)$ denotes the sigmoid function; ⊙ denotes element‐wise multiplication; $f^{7\times 7}\left(\cdot \right)$ denotes a 7×7 convolution; and (;) denotes channel‐wise concatenation.

The CBAM module markedly strengthens the model's sensitivity to renal contours, morphological abnormalities and regions with intensity variations, thereby improving overall discriminative robustness.

### 2.4 Model Training and Validation

#### 2.4.1 Data Split and Preprocessing

The dataset comprised 346 DMSA scans, including 170 VUR‐positive and 176 VUR‐negative cases. The full dataset was first randomly divided into a development cohort (90%) and an independent test cohort (10%) using a 9:1 ratio. The independent test cohort was not used for model fitting or fold assignment during cross‐validation.

Within the development cohort, 10‐fold cross‐validation was performed. Specifically, the development cohort was partitioned into 10 approximately equal folds. In each iteration, 9 folds were used for training and the remaining fold was used for validation, so that each sample in the development cohort served as validation data exactly once. This procedure was used to assess model stability and performance across folds while maximising data utilisation within the development cohort.

#### 2.4.2 Training Data Preparation

During training and evaluation, DMSA data were organised under both the severe‐side and bilateral‐side settings. For the severe‐side setting, one kidney was selected according to the predefined side‐selection rule described in Section 2.2.4, whereas for the bilateral‐side setting, the left and right kidneys were processed separately and later aggregated at the patient level. This strategy ensured that the input data contained representative lesion‐related features, thereby facilitating improved prediction performance.

#### 2.4.3 Model Optimisation

The Swin Transformer–based model was trained using the AdamW optimiser [23, 24] (initial learning rate = 0.0001; weight decay = 0.01), cross‐entropy loss (CrossEntropyLoss) and a batch size of 3. Training was conducted under 10‐fold cross‐validation, with performance evaluated on the validation set for each fold. Early stopping was applied [25, 26, 27]: training was terminated if validation performance did not improve for 20 consecutive epochs.

For the comparative experiments, all backbone models, including Swin Transformer, ResNet34, DenseNet121, DenseNet161, GoogLeNet and MaxViT, were implemented under the same experimental framework as MedSwinNet. Specifically, all models used pretrained weights and their original classification heads were uniformly replaced with task‐specific binary classification heads. In addition, all comparison models shared the same data split strategy, preprocessing pipeline, input size, data augmentation scheme, loss function, optimiser, batch size, early stopping criterion and evaluation protocol. A unified training script was used across experiments, with only the backbone architecture being replaced.

#### 2.4.4 Testing and Evaluation

At evaluation, we assessed performance under both the severe‐side and bilateral‐side settings. For the bilateral‐side setting, the left and right kidneys were predicted separately and then aggregated at the patient level: if both sides were predicted as negative, the case was labelled negative; otherwise, it was labelled positive.

#### 2.4.5 Performance Metrics

Model performance was assessed using loss, AUC, accuracy, sensitivity and specificity under both the severe‐side and bilateral‐side settings.

For the validation results, metrics were calculated on the validation fold in each cross‐validation iteration and then averaged across the 10 folds.

For the test results, after each fold‐specific training process, the resulting model was additionally evaluated on the same held‐out independent test cohort. Therefore, the reported test metrics represent the average performance of the 10 fold‐specific models on the independent test cohort, rather than a one‐time evaluation of a single final model. This evaluation strategy was adopted to provide a more stable estimate of model performance under the present experimental design.

For the main test metrics, 95% confidence intervals (CIs) were calculated from the 10 fold‐specific performance estimates on the independent test cohort using the t distribution.

### 2.5 Loss Function Selection and Comparative Analysis

For the binary classification task, we compared two commonly used loss functions—CrossEntropyLoss and BCEWithLogitsLoss [28, 29]—in our setting.

CrossEntropyLoss: suitable for classification, producing normalised probabilities via softmax and explicitly modelling mutual exclusivity between classes; it typically converges faster and yields better final classification performance.

BCEWithLogitsLoss: appropriate for binary or multi‐label tasks by combining a sigmoid activation with binary cross‐entropy; however, it models classes independently, which may underutilise class exclusivity and may be slightly inferior under balanced class distributions.

Under the same model (MedSwinNet) and hyperparameter settings, CrossEntropyLoss outperformed BCEWithLogitsLoss in terms of AUC, accuracy and convergence behaviour. Training was more stable and the final decision boundary was clearer. Therefore, CrossEntropyLoss was selected as the default loss function to optimise VUR prediction performance.

## 3 Results

In this study, we developed MedSwinNet, a Swin Transformer–based model enhanced with multi‐scale feature extraction, CBAM attention, and a gated mechanism and achieved notable performance improvements for paediatric vesicoureteral reflux (VUR) prediction. The experimental results and analyses are presented below.

### 3.1 Visualisation of Preprocessing Effects

**FIGURE 4:**
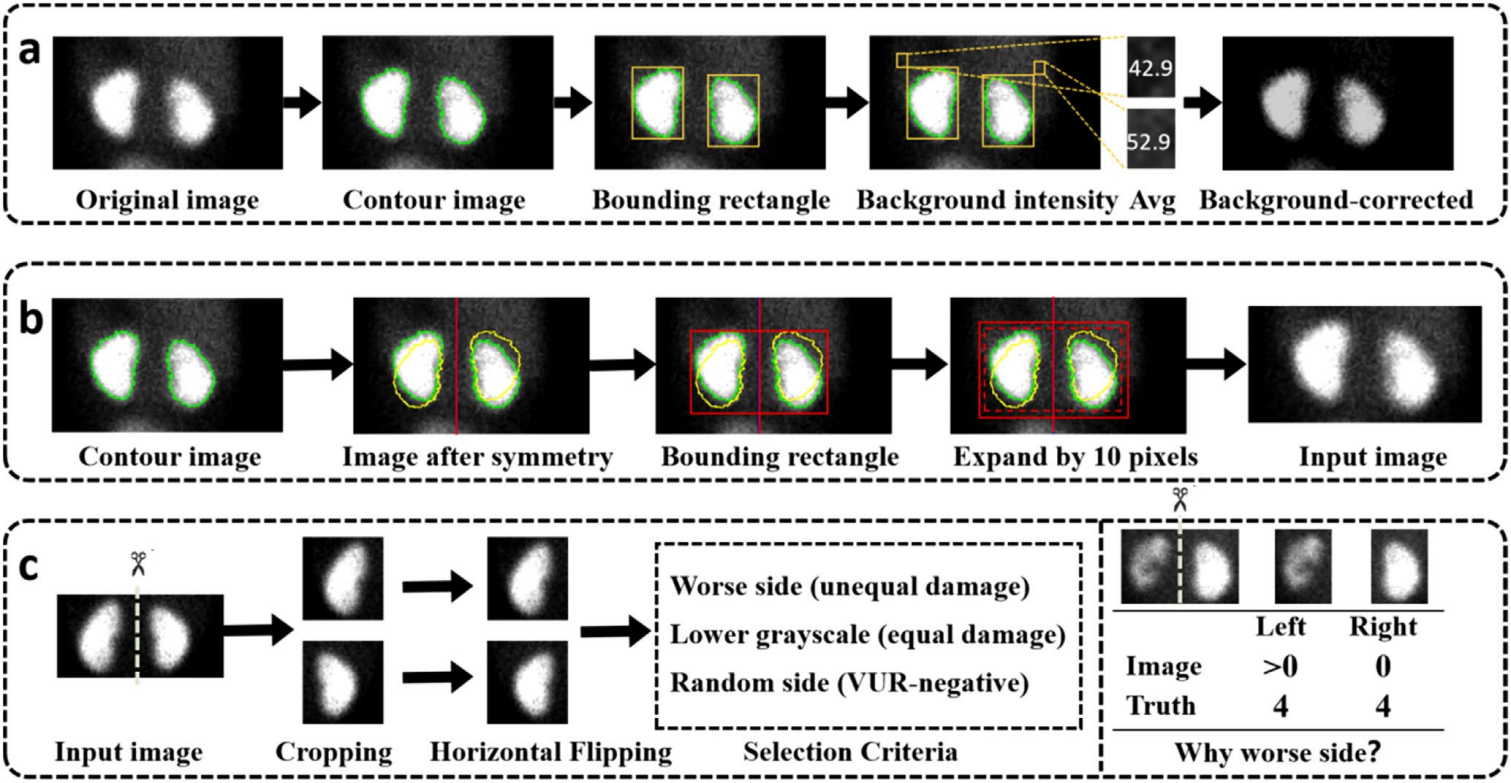
**Illustration of the DMSA preprocessing pipeline: Background subtraction, automatic threshold‐based segmentation and severe‐side selection.** (A) Background correction: Integral background subtraction is applied to the raw scan to reduce the influence of uptake outside the kidneys and to enhance contrast between the renal cortex and background. (B) ROI extraction and scale normalisation: Bilateral kidney contours are delineated using Otsu's automatic thresholding; a midline is computed and symmetric extension is performed to obtain a complete bilateral renal outline. The bilateral renal region is then cropped using the minimum bounding rectangle enclosing both kidneys (with ≥ 10‐pixel margins on all sides) and uniformly resized to 224 × 448 pixels. (C) Severe‐side selection strategy: For the severe‐side setting, one kidney was selected from each bilateral DMSA scan. When bilateral VUR grades were different, the kidney with the higher VUR grade was selected. When bilateral VUR grades were identical, the kidney with the lower mean tracer uptake (lower mean intensity) was selected. For VUR‐negative cases, one side was randomly selected using a fixed random seed to ensure reproducibility. This side‐selection procedure was implemented programmatically across the dataset.

Figure 4 illustrates representative examples of the DMSA preprocessing pipeline. Figure 4A shows a raw scan and the corresponding background‐corrected result. After integral background subtraction, radiotracer uptake outside the kidneys is markedly attenuated, thereby increasing the contrast between the renal cortex and surrounding background. Figure 4B demonstrates the key preprocessing steps, including automatic delineation of bilateral kidney contours using Otsu's method, midline estimation and symmetric extension, cropping with a bounding rectangle and resizing to 224 × 448 pixels. This procedure preserves complete bilateral renal anatomy while removing irrelevant regions such as the liver and spleen.

The right panel of Figure 4C illustrates the severe‐side selection strategy. For the severe‐side setting, one kidney was selected from each bilateral DMSA scan according to a predefined programmatic rule. Specifically, when bilateral VUR grades differed, the kidney with the higher VUR grade was selected. When bilateral VUR grades were identical, the kidney with the lower mean intensity was selected. For VUR‐negative cases, one side was randomly selected using a fixed random seed. This design was intended to provide a lesion‐focused input for model training while maintaining a reproducible side‐selection pipeline.

### 3.2 Image Quality Control (QC) and Consistency Across Data Splits

**FIGURE 5:**
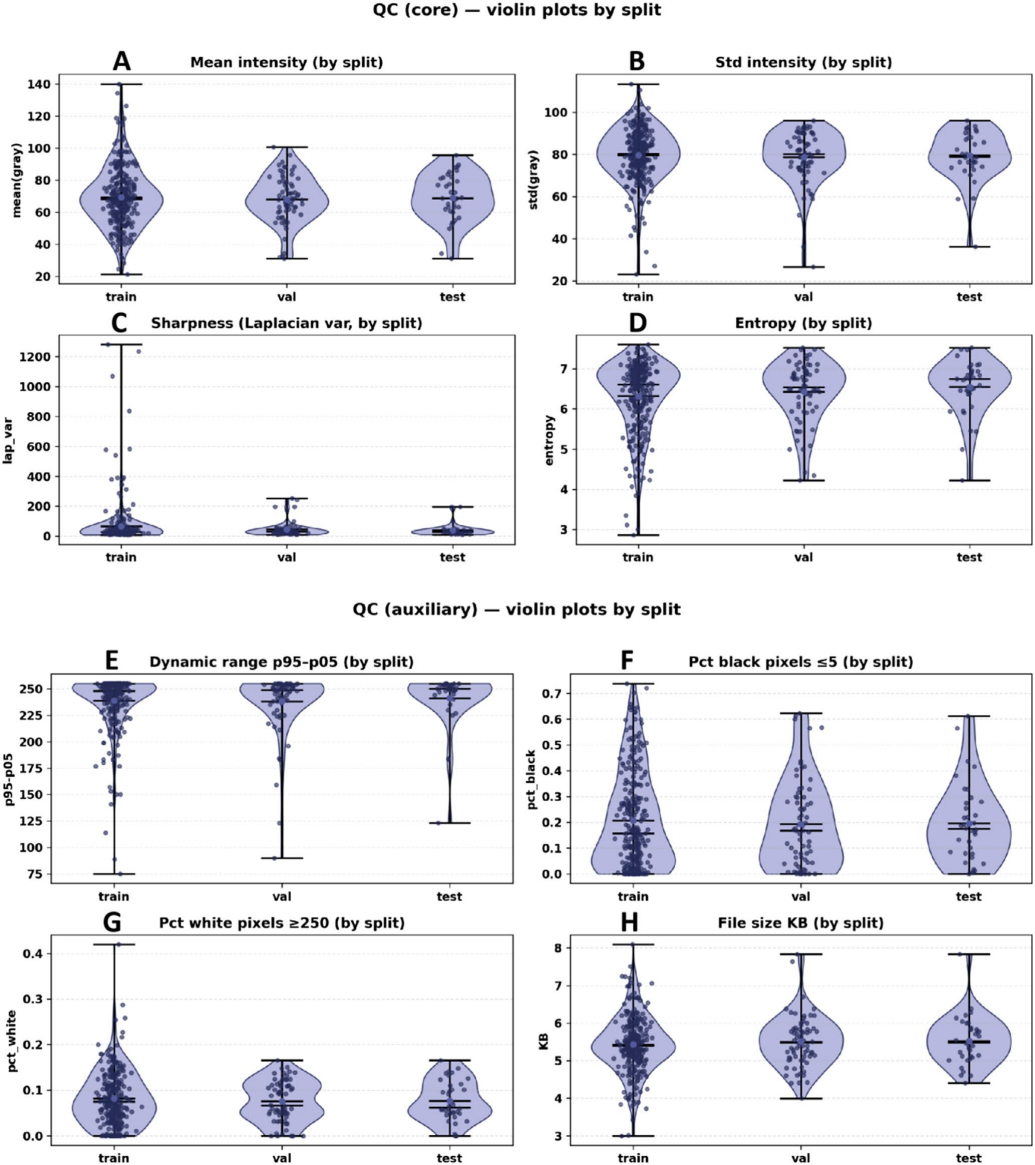
**Distributions of image quality control (QC) metrics across the training, validation and test sets**. Violin plots depict the distributions of image‐level QC metrics for different data splits (train/val/test). Each dot represents the value from a single image, and the internal horizontal lines/error bars denote the summary statistics displayed within each panel (mean/median and range, as indicated in the plot). The upper panel shows core QC metrics: (A) mean intensity (mean grey), (B) standard deviation of intensity (std grey), (C) sharpness (Laplacian variance) and (D) entropy. The lower panel shows auxiliary QC metrics: (E) intensity dynamic range (p95–p05), (F) black‐pixel clipping ratio (proportion of pixels ≤ 5), (G) white‐pixel saturation ratio (proportion of pixels ≥ 250) and (H) file size (KB). Overall, the distributions are highly overlapping across train/val/test for all metrics, suggesting no evident QC‐related distribution shift between splits and supporting the comparability of subsequent model performance evaluations.

To exclude potential evaluation bias caused by image quality differences between the training/validation/test sets and to verify split comparability, we computed and compared multiple quality control (QC) metrics across all images, including mean intensity (Mean grey), intensity standard deviation (Std grey), dynamic range (p95–p05), sharpness (Laplacian variance), entropy, proportion of near‐black pixels (Pct black ≤ 5), proportion of saturated pixels (Pct white ≥ 250) and file size. QC distributions were visualised using violin plots (Figure 5) and summarised as median [IQR] in the aggregated QC table. In addition, Mann–Whitney *U* (MWU) and Kolmogorov–Smirnov (KS) tests were performed to compare the training and test sets (Table 1).

**TABLE 1:** Distribution shift tests for QC metrics.

Metric	Train median [IQR]	Val median [IQR]	Test median [IQR]	P (MWU, train vs. test)	P (KS, train vs. test)
Mean grey	68.162 [57.692, 79.046]	67.957 [60.60, 78.828]	68.735 [60.437, 81.234]	0.792	0.731
Std grey	80.312 [73.227, 88.179]	80.035 [73.672, 89.105]	78.780 [74.434, 88.588]	0.971	0.827
Dyn range (p95–p05)	248.000 [235.00, 254.00]	249.000 [238.00, 254.00]	250.000 [244.750, 254.0]	0.565	0.183
Sharpness (Lap var)	27.271 [20.502, 43.226]	30.452 [20.177, 44.227]	28.499 [19.447, 37.804]	0.621	0.802
Entropy	6.609 [5.957, 6.948]	6.542 [6.056, 6.952]	6.747 [6.385, 6.944]	0.258	0.23
Pct black ≤ 5	0.156 [0.044, 0.324]	0.167 [0.044, 0.297]	0.174 [0.080, 0.276]	0.849	0.726
Pct white ≥ 250	0.075 [0.040, 0.114]	0.066 [0.049, 0.111]	0.062 [0.049, 0.111]	0.849	0.799
File size (KB)	5.405 [5.045, 5.898]	5.485 [5.096, 5.837]	5.485 [5.115, 5.848]	0.578	0.761

As shown in Figure 5, the distributions of most QC metrics overlap substantially across the training, validation and test sets. The upper part of the combined Figure 5 (Figure 5A–D) presents core metrics (Mean grey, Std grey, Sharpness and Entropy), for which the three splits exhibit highly similar distributional shapes, with only a few outliers and no systematic shifts. The lower part (Figure 5E–H) shows auxiliary metrics (dynamic range, Pct black, Pct white and file size), which also display strong overlap and do not suggest that any split is systematically more ‘overexposed/underexposed,’ lower in contrast or markedly different in export quality.

Table 1 provides quantitative confirmation. Median QC levels are comparable across Train/Val/Test (e.g., Mean grey ≈68, Std grey ≈79–80, dynamic range ≈248–250, Entropy ≈6.5–6.7; black/white clipping ratios are of similar magnitude). Moreover, for all included QC metrics, MWU and KS tests between the training and test sets did not reach statistical significance (*p* > 0.05; Table 1), indicating no detectable distribution shift attributable to image quality. Collectively, these results support good consistency across data splits in terms of brightness, contrast, sharpness, information content, exposure/clipping and file size, thereby strengthening the credibility of subsequent performance comparisons and generalisation assessments.

### 3.3 Model Performance Evaluation

**FIGURE 6:**
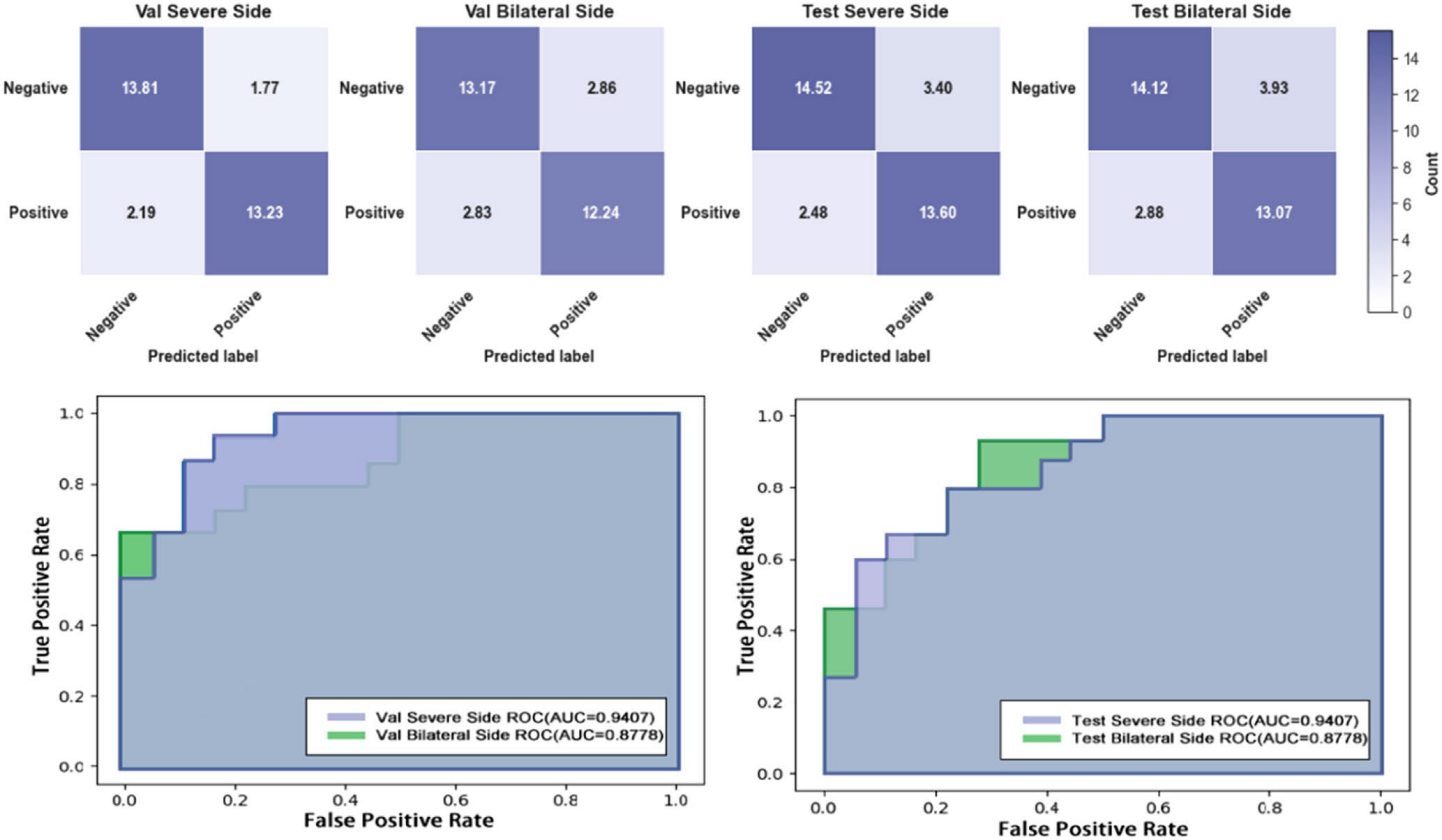
**Confusion matrices and ROC curves under different evaluation settings.** The upper row shows confusion matrices for four settings: Validation severe‐side (Val severe‐side), validation bilateral‐side (Val bilateral‐side), test severe‐side (Test severe‐side) and test bilateral‐side (Test bilateral‐side). The *x*‐axis denotes predicted classes and the *y*‐axis denotes ground‐truth classes; colour intensity reflects the number of samples. When decimal values are shown, they represent confusion‐matrix counts averaged across multiple folds. The lower row shows the corresponding ROC curves: The left panel compares ROC curves for severe‐side versus bilateral‐side on the validation set, and the right panel compares ROC curves for severe‐side versus bilateral‐side on the test set. The area under the curve (AUC) summarises overall discriminative performance across decision thresholds.

After completing preprocessing and verifying split consistency, we evaluated the classification performance of the proposed model on the validation and test sets. Figure 6 presents confusion matrices and ROC curves under different task settings, providing complementary threshold‐dependent and threshold‐independent assessments of discriminative performance.

As shown in Figure 6, the model demonstrates good discrimination for both the severe‐side and bilateral‐side prediction tasks on the validation and test sets. The ROC curves lie well above the diagonal line of a random classifier and show stable shapes, indicating strong separability between positive and negative cases. In terms of AUC, the validation severe‐side and bilateral‐side tasks achieved AUCs of 0.9407 and 0.8778, respectively, while the test severe‐side and bilateral‐side tasks achieved AUCs of 0.8630 and 0.8556. These results suggest that the severe‐side setting performs better overall than the bilateral‐side setting, and that the model maintains favourable generalisation on the test set.

The confusion matrices further characterise error patterns under each setting. Overall, the model achieves high proportions of correct classifications for both negative and positive cases, without obvious extreme bias on the test set (e.g., a strong tendency to predict only positives or only negatives). Compared with the bilateral‐side task, the severe‐side task shows more stable recognition of positive cases, consistent with the ROC/AUC findings. This supports the rationale that selecting the ‘severe‐side’ kidney helps emphasise more discriminative lesion‐related cues, thereby improving classification performance.

Collectively, Figure 6 demonstrates the effectiveness and potential generalisability of the proposed model for VUR prediction, with particularly strong discriminative capability under the severe‐side setting.

To improve the statistical interpretability of the performance results, we further summarised the main test metrics with 95% confidence intervals. On the independent test cohort, the severe‐side setting achieved an AUC of 0.863 (95% CI, 0.832–0.894), an accuracy of 0.829 (95% CI, 0.801–0.857), a sensitivity of 0.846 (95% CI, 0.809–0.883) and a specificity of 0.810 (95% CI, 0.772–0.848). Under the bilateral‐side setting, the corresponding values were an AUC of 0.856 (95% CI, 0.821–0.890), an accuracy of 0.800 (95% CI, 0.766–0.834), a sensitivity of 0.819 (95% CI, 0.781–0.857) and a specificity of 0.782 (95% CI, 0.743–0.821).

### 3.4 Ablation and Comparative Experiments

#### 3.4.1 Ablation Experiments

**FIGURE 7:**
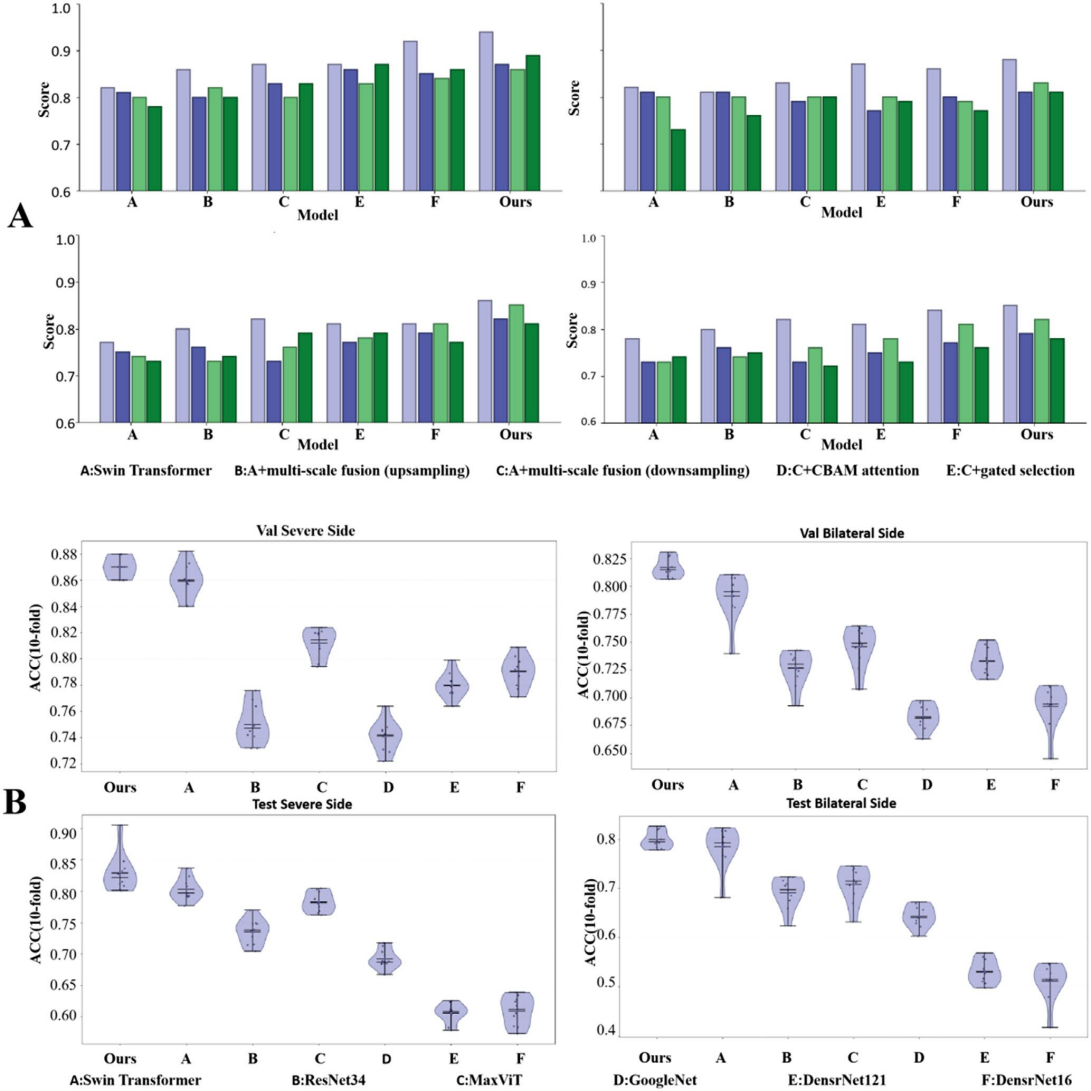
**Visualisation of ablation and comparative experiments.** Ablation study: Bar charts compare multi‐metric performance across different model configurations under four settings (validation/test × severe‐side/bilateral‐side). Metrics include AUC, *F*1‐score, sensitivity and specificity (as indicated in the legend). Models A–E represent incremental ablation variants: A, baseline Swin Transformer; B, multi‐scale feature fusion with unified upsampling‐based fusion; C, multi‐scale feature fusion with unified downsampling‐based fusion; D, model C plus CBAM attention; E, model C plus gated selection; Ours, the full MedSwinNet (model D plus gated selection). Comparative study: Violin plots show accuracy distributions of MedSwinNet versus several mainstream backbone networks across the four evaluation settings (Val/Test × severe‐side/bilateral‐side), reflecting fold‐to‐fold variability and stability. Internal markers denote central tendency and dispersion (as displayed in the figure). Overall, MedSwinNet achieves consistently higher performance with more stable distributions across datasets and input configurations.

To quantify the contribution of each proposed component, we conducted ablation experiments (Table 2) to evaluate the effects of different feature fusion strategies, attention mechanisms and gating structures. The upper part of Figure 7 further visualises AUC, *F*1‐score, sensitivity and specificity across ablation configurations.

**TABLE 2:** Ablation study results.

Method	Train	Validation		Test	
	Severe‐side	Severe‐side	Bilateral‐side	Severe‐side	Bilateral‐side
A	0.8918	0.8603	0.7913	0.8043	0.7851
B	0.8834	0.8614	0.7901	0.8003	0.7864
C	0.8998	0.8674	0.7961	0.8064	0.7866
D	0.9091	0.8699	0.7973	0.8116	0.7990
E	0.9116	0.8681	0.7986	0.8161	0.7981
MedSwinNet	0.9286	0.8723	0.8170	0.8290	0.7997

As shown in Table 2, the baseline Model A (original Swin Transformer) achieved validation severe‐side/bilateral‐side accuracies of 0.8603/0.7913 and test severe‐side/bilateral‐side accuracies of 0.8043/0.7851. Incorporating multi‐scale feature fusion improved performance: unified fusion via upsampling (Model B) yielded validation accuracies of 0.8614/0.7901, while unified fusion via downsampling (Model C) further improved to 0.8674/0.7961, suggesting that downsampling‐based fusion better integrates deep semantic features with shallow details for enhanced discrimination.

Adding the CBAM attention module on top of Model C (Model D) increased validation severe‐side/bilateral‐side accuracies to 0.8699/0.7973 and test severe‐side/bilateral‐side accuracies to 0.8116/0.7990, indicating that attention can strengthen focus on lesion‐relevant regions and improve discrimination, particularly in the bilateral‐side setting. Further introducing the gating mechanism on top of Model C (Model E) increased test severe‐side accuracy to 0.8161 (higher than Model D at 0.8116) and improved validation bilateral‐side accuracy to 0.7986 (higher than Model D at 0.7973), suggesting that gating facilitates adaptive cross‐level feature selection and denoising. Meanwhile, Model E achieved a test bilateral‐side accuracy of 0.7981, comparable to Model D (0.7990), implying that attention and gating may confer slightly different gains for the bilateral‐side task.

The full MedSwinNet model achieved the best overall performance among all configurations: training severe‐side accuracy of 0.9286; validation severe‐side/bilateral‐side accuracies of 0.8723/0.8170; and test severe‐side/bilateral‐side accuracies of 0.8290/0.7997 (Table 2). Consistent with the multi‐metric visualisation in Figure 7A, model performance generally improved as components were progressively introduced, validating the synergistic benefits of multi‐scale fusion, CBAM attention and gating and suggesting improved generalisation and potential clinical utility.

#### 3.4.2 Comparison With Mainstream Models

To further assess the relative advantage of MedSwinNet, we compared it with several widely used deep learning backbones under a unified experimental setting (Table 3), including Swin Transformer, ResNet34 [30], DenseNet121 and DenseNet161 [31], GoogLeNet [32] and MaxViT [33]. Figure 7B visualises performance distributions across models using violin plots under four settings (Val/Test × severe‐side/bilateral‐side).

**TABLE 3:** Comparative study results.

Method	Train	Validation		Test	
	Severe‐side	Severe‐side	Bilateral‐side	Severe‐side	Bilateral‐side
MedSwinNet	0.9286	0.8723	0.8170	0.8290	0.7997
Swin transformer	0.8918	0.8603	0.7913	0.8043	0.7851
ResNet34	0.8214	0.7552	0.7267	0.7333	0.6915
MaxViT	0.8353	0.8109	0.7458	0.7827	0.7088
GoogLeNet	0.7945	0.7455	0.6828	0.6943	0.6434
DenseNet121	0.7810	0.7857	0.7331	0.6057	0.5315
DenseNet161	0.8004	0.7889	0.6924	0.6082	0.5110

MedSwinNet achieved the best performance across both severe‐side and bilateral‐side tasks on the validation and test sets: 0.8723/0.8170 on validation and 0.8290/0.7997 on test (Table 3). In comparison, the Swin Transformer baseline achieved 0.8043/0.7851 on the test set (severe‐side/bilateral‐side), ResNet34 achieved 0.7333/0.6915 and MaxViT achieved 0.7827/0.7088, with other models performing lower overall. The lower part of Figure 7 further shows that MedSwinNet exhibits an overall higher performance distribution, indicating more stable and reliable generalisation across task settings. Together, these results demonstrate the robustness advantage of MedSwinNet for modelling subtle cues in DMSA‐based VUR classification.

### 3.5 Clinician Interpretation and Descriptive Comparison With Model Performance

To improve the clinical interpretability of the results, we further incorporated clinician readings and performed descriptive analyses for both the single‐kidney and bilateral‐kidney tasks, with a reference comparison against the model's confusion‐matrix–based results on the test set. Notably, the ‘single‐kidney’ clinician assessment is not fully equivalent to the model's severe‐side input definition, as the kidney‐side selection rules differ between clinician reading and severe‐side selection. Therefore, the ‘clinician vs. model’ comparison in this section is intended for descriptive reference rather than paired statistical inference at the individual‐case level.

As shown in Table 4, for the clinician single‐kidney task, the true positive rate was 28.44%, whereas the clinician‐predicted positive rate was 50.30%, indicating a tendency towards ‘positive’ calls. The confusion matrix was TN = 278, FP = 200, FN = 54 and TP = 136. Clinician performance in the single‐kidney task was: accuracy = 0.620, sensitivity = 0.716, specificity = 0.582, precision = 0.405, *F*1‐score = 0.517 and Cohen's kappa = 0.241. Overall, clinicians demonstrated relatively high sensitivity at the cost of a high false‐positive rate, resulting in lower specificity and precision.

**TABLE 4:** Clinician assessment results and descriptive comparison with the model.

Metric	Clinician (single‐side)	Clinician (bilateral‐side)	Model (severe‐side)	Model (bilateral‐side)
Accuracy	0.620	0.705	0.829	0.800
Sensitivity	0.716	0.653	0.846	0.819
Specificity	0.582	0.759	0.810	0.782
Precision	0.405	0.737	0.800	0.769
*F*1‐score	0.517	0.693	0.822	0.793
Cohen's kappa	0.241	0.411	—	—

For the clinician bilateral‐kidney task, the true positive rate was 50.87% and the clinician‐predicted positive rate was 45.09%, reflecting a more conservative tendency than in the single‐kidney task. The confusion matrix was TN = 129, FP = 41, FN = 61 and TP = 115. Clinician performance in the bilateral task was: accuracy = 0.705, sensitivity = 0.653, specificity = 0.759, precision = 0.737, *F*1‐score = 0.693 and Cohen's kappa = 0.411. Compared with the single‐kidney task, clinicians achieved higher specificity, precision, *F*1‐score and agreement (*κ*), but lower sensitivity, indicating a more cautious decision strategy that reduced false positives while increasing missed positives.

We further performed a descriptive reference comparison between clinician results and the model's averaged confusion matrices on the test set. For the Test severe‐side task, the model's reference metrics were accuracy = 0.829, sensitivity = 0.846, specificity = 0.810, precision = 0.800 and *F*1‐score = 0.822; for the Test bilateral‐side task, the corresponding reference metrics were accuracy = 0.800, sensitivity = 0.819, specificity = 0.782, precision = 0.769 and *F*1‐score = 0.793. Overall, the model achieved higher composite metrics than the clinician baseline in both tasks, with a more pronounced improvement in the severe‐side setting. These findings suggest that the proposed model may provide valuable decision support for complex pattern recognition in clinical practice.

It should be noted that model evaluation in this study was based on a two‐stage design consisting of a 9:1 split into a development cohort and an independent test cohort, followed by 10‐fold cross‐validation within the development cohort. Validation results were obtained by averaging performance across the 10 validation folds. In addition, after each fold‐specific training process, the corresponding model was evaluated on the same held‐out test cohort and the reported test results represent the average performance of the 10 fold‐specific models on that independent test cohort. Accordingly, the model results reported in this study are based on fold‐wise aggregated evaluation rather than on a single one‐time final‐model prediction set strictly aligned with clinician case‐level readings. For this reason, the ‘clinician vs. model’ comparison presented here should be interpreted as descriptive rather than as a rigorous paired statistical comparison.

## 4 Discussion

### 4.1 Main Findings and Clinical Implications

This study established an AI‐enabled prediction framework for VUR using static renal DMSA scintigraphy in children and proposed an enhanced model, MedSwinNet, to improve discrimination under weak‐signal and complex‐background conditions. Overall, our results indicate: (i) the model achieved favourable discriminative performance on both the validation and test sets, with relatively stable outputs under the severe‐side and bilateral‐side settings (Figure 6); (ii) ablation experiments confirmed the practical contributions of multi‐scale feature fusion, CBAM attention and the gating mechanism to performance gains (Figure 7A and Table 2); (iii) comparative experiments further demonstrated that MedSwinNet outperformed or remained consistently competitive against widely used backbones including ResNet34, DenseNet, GoogLeNet and MaxViT, with improved fold‐to‐fold stability (Figure 7B and Table 3).

From the perspective of the clinical pathway, VCUG/MCU remains the gold standard for VUR diagnosis and grading but is invasive and imposes substantial burden on paediatric comfort and compliance. In contrast, DMSA scintigraphy is routinely used to assess cortical involvement and scarring risk. The potential clinical value of our approach lies in leveraging DMSA‐derived phenotypic information related to cortical injury for risk stratification, providing quantitative support for the decision of whether VCUG/MCU is warranted. This may help maintain sensitivity for identifying high‐risk individuals while reducing unnecessary invasive testing.

### 4.2 Rationale and Implications of Severe‐Side Training With Bilateral‐Side Evaluation

We adopted a setting in which training was performed using severe‐side inputs only, while both severe‐side and bilateral‐side settings were evaluated during validation and testing. The main motivation for this design was to improve the learning efficiency for lesion‐relevant signals while still providing a complementary assessment under a setting that more closely resembles clinical interpretation.

It should be noted that the severe‐side setting in this study was label‐informed. Specifically, when bilateral VUR grades differed, the kidney with the higher VUR grade was selected; when bilateral VUR grades were identical, the kidney with the lower mean intensity on DMSA was selected; and for VUR‐negative cases, one side was randomly selected using a fixed random seed. Therefore, the severe‐side setting should be interpreted as a lesion‐focused research strategy designed to direct model training towards the more severely affected renal phenotype, rather than as a fully deployment‐equivalent clinical workflow.

From a modelling perspective, this strategy was intended to reduce the dilution effect that may arise when bilateral inputs contain one relatively less affected side and one more affected side, thereby allowing the network to focus more effectively on lesion‐relevant phenotypic signals. This may help improve feature learning efficiency and enhance discrimination of subtle abnormal patterns in DMSA scans. However, because severe‐side selection used the reference‐standard VUR grading when bilateral grades differed, this setting may introduce potential reference‐standard‐related bias.

For this reason, we additionally evaluated the model under the bilateral‐side setting, which provides a more complementary and deployment‐relevant assessment. The differences observed between severe‐side and bilateral‐side results further suggest that bilateral‐side prediction is a more challenging but clinically important scenario, and future work should explore fully image‐driven or clinically deployable side‐selection strategies.

### 4.3 Structural Contributions: Synergistic Effects of Multi‐Scale Fusion, Attention and Gating

The ablation study (Figure 7A and Table 2) showed a consistent upward trend across multiple evaluation metrics as modules were progressively introduced, indicating clear incremental benefits. First, multi‐scale feature fusion enables simultaneous capture of shallow texture details and deep semantic structure, which is well suited to the DMSA pattern of ‘focal defects coexisting with global morphology.’ In our experiments, unified fusion via downsampling was more stable and yielded larger gains than fusion via upsampling. A plausible explanation is that upsampling interpolation in low‐resolution, low‐contrast inputs may introduce additional noise or artefacts [34], whereas downsampling facilitates cross‐level alignment and robust integration, thereby strengthening lesion‐region representation.

Second, the CBAM attention module enhances lesion‐relevant responses through channel and spatial pathways. Under intensity inhomogeneity, substantial background noise or blurred boundaries, CBAM suppresses nonspecific interference and improves focus on subtle pathological cues, which is particularly beneficial for stabilising discrimination under the bilateral‐side setting.

Finally, the gating mechanism adaptively reweights and selects cross‐level/cross‐scale features, dynamically suppressing feature sources dominated by noise on a per‐sample basis and improving adaptability across variable‐quality inputs. Taken together, multi‐scale fusion provides complementary information, attention strengthens salient regions and gating enables adaptive selection. This synergistic chain helps explain why the full MedSwinNet consistently outperforms partial configurations across multiple evaluation settings.

### 4.4 Comparison With Mainstream Methods and Discussion of Fold‐To‐Fold Stability

Comparative experiments (Figure 7B and Table 3) showed that MedSwinNet achieved superior or consistently competitive performance across the four settings (Val/Test × severe‐side/bilateral‐side), with a more favourable distributional profile (higher performance levels with controlled dispersion). In medical imaging, stability and reproducibility are closely linked to clinical usability; models that are highly sensitive to data perturbations or case variability may exhibit unpredictable performance at deployment. The fold‐wise distributions visualised by violin plots provide intuitive evidence of stability, suggesting that MedSwinNet not only improves mean performance but also enhances robustness during training and evaluation.

Methodologically, while the Swin Transformer backbone offers hierarchical representation and window‐based attention, the DMSA setting—characterised by weak signals, substantial noise and focal lesions—demands stronger multi‐scale integration and denoising. By augmenting the backbone with multi‐scale fusion, attention‐based recalibration and gated selection, MedSwinNet is better tailored to this modality, which is reflected in its comparative advantage.

### 4.5 Support From QC Consistency for the Credibility of Performance Evaluation

In medical imaging studies, quality drift across training/validation/test sets (e.g., differences in brightness, contrast, sharpness or clipping/saturation) can lead models to exploit distributional discrepancies rather than pathological cues, thereby inflating apparent ‘generalization performance.’ To mitigate such bias, we conducted a systematic QC assessment (Figure 5 and Table 1), including core metrics (mean intensity, intensity standard deviation, sharpness, entropy) and auxiliary metrics (dynamic range, black‐pixel clipping ratio, white‐pixel saturation ratio and file size). The results demonstrated substantial overlap across splits for all QC metrics, and statistical testing did not identify significant differences (Table 1). Therefore, subsequent performance comparisons are more likely to reflect methodological improvements rather than spurious advantages driven by quality drift, strengthening the internal validity and reliability of our conclusions.

### 4.6 Clinician Reading Patterns and the Potential Value of Model‐Assisted Diagnosis

The clinician reading results added in this study provide a clinically relevant baseline for interpreting model performance. The results indicate that, in the clinician single‐kidney task, the reading strategy was characterised by ‘high sensitivity and low specificity,’ that is, a tendency to classify more cases as positive to reduce the risk of missed diagnoses. This approach has practical clinical rationale in paediatric VUR screening, as missed cases may delay further examinations and interventions; however, it also leads to a higher false‐positive rate and may increase unnecessary follow‐up testing. Consistent with this pattern, the clinician‐predicted positive rate in the single‐kidney task (50.30%) was markedly higher than the true positive proportion (28.44%).

In contrast, clinician performance in the bilateral‐kidney task was more conservative: specificity and precision improved, whereas sensitivity decreased. This suggests that when interpreting bilateral presentations, clinicians may rely more heavily on clearer imaging cues before calling a case positive, thereby reducing false positives but increasing false negatives. These findings highlight how task definitions and presentation formats can influence human decision strategies, and they also indicate that a single metric (e.g., accuracy) is insufficient to fully characterise clinical reading quality. Instead, sensitivity, specificity, *F*1‐score and agreement metrics should be considered jointly.

In the descriptive comparison with the model, the model achieved higher composite metrics than the clinician baseline for both single‐kidney and bilateral‐kidney tasks, suggesting advantages in feature extraction stability and decision consistency. Considering the design of MedSwinNet (multi‐scale feature fusion, CBAM attention and gated selection), this advantage may stem from the model's ability to integrate local and global information more consistently under complex background, low‐contrast or blurred‐boundary conditions, thereby reducing variability driven by subjective experience. Importantly, the current comparison is based on fold‐aggregated averaged confusion matrices rather than per‐case model outputs; therefore, these findings should be interpreted as evidence of ‘potential value for decision support,’ rather than definitive proof of statistically significant superiority.

From a clinical implementation perspective, the model is not necessarily intended to replace clinicians but may serve as an assistive tool: under a high‐sensitivity screening strategy, it may help reduce unnecessary false positives; under a conservative reading strategy, it may help identify potential missed positives. Future work incorporating multicentre data, stratified analyses by clinician seniority and per‐case probability outputs would help clarify the model's incremental benefit across clinical scenarios and facilitate the establishment of a human–AI collaborative reading workflow.

### 4.7 Limitations

First, this study was based on a single‐centre retrospective cohort, which may introduce selection bias and limit the representativeness of the study population. Although QC analyses supported consistency across data splits, the model was developed and evaluated within a relatively consistent institutional workflow and its performance may therefore be influenced by centre‐specific factors such as patient spectrum, imaging acquisition protocols and clinical testing pathways. Accordingly, multicentre external validation and prospective evaluation will be necessary to further assess robustness and generalisability before broader clinical deployment.

In addition, the severe‐side input strategy was label‐informed when bilateral VUR grades differed and therefore may have introduced potential reference‐standard‐related bias. Although this setting was designed to facilitate lesion‐focused learning, it does not fully reflect a deployment‐equivalent clinical workflow. For this reason, we additionally reported bilateral‐side evaluation results as a complementary and more practically relevant assessment.

### 4.8 Future Directions

Future work can be advanced in four directions. First, multicentre external validation should be conducted to evaluate generalisability across devices and protocols and domain adaptation strategies may be explored accordingly; in addition, external validation should be reported systematically in accordance with TRIPOD+AI and related reporting guidance [35]. Second, calibration assessment (e.g., calibration curves and Brier score) [36] and decision curve analysis (DCA) [37] should be incorporated to quantify net clinical benefit and to evaluate the practical value of the model for triaging decisions regarding VCUG/MCU. Although 95% confidence intervals were added for the main test metrics in the current revision, calibration‐related evaluation was not formally incorporated and should be addressed in future work before broader clinical deployment. Third, explainability approaches such as Grad‐CAM combined with expert annotations should be used to establish region‐level evidence chains and enhance clinical interpretability and trust. Fourth, multimodal risk modelling that integrates imaging with clinical variables (e.g., age, history of recurrent UTI, ultrasound abnormalities) should be explored to better align with real‐world workflows; relevant methodological insights on multi‐source fusion and interpretable modelling can be drawn from multimodal multi‐granularity fusion and heterogeneous graph attention approaches in biomedical prediction tasks [38, 39, 40]. In addition, future work may consider incorporating physiological signals [41] (e.g., vital signs) and multi‐omics evidence [42, 43] (e.g., proteomics and spatial transcriptomics) and linking these data to dynamical modelling of programmed cell‐death networks to further strengthen mechanistic interpretability and translational value [44].

## 5 Conclusion

This study proposed and validated MedSwinNet, an intelligent approach for predicting vesicoureteral reflux (VUR) based on static renal DMSA scintigraphy in children. Using VCUG/MCU findings as gold‐standard labels, we constructed a clinical dataset from the Second Affiliated Hospital of Wenzhou Medical University and implemented a complete pipeline encompassing preprocessing, severe‐side selection, model training and performance evaluation. Quality control (QC) analyses showed that the training, validation and test sets were broadly consistent in key metrics—including brightness, contrast, sharpness, dynamic range and clipping ratios—supporting split comparability and the credibility of subsequent results.

Experimental results demonstrated that MedSwinNet achieved stable and competitive predictive performance on both the validation and test sets. Although training was performed exclusively with severe‐side inputs, the model maintained good generalisation under both severe‐side and bilateral‐side evaluation settings. Ablation experiments further confirmed the synergistic benefit of multi‐scale feature fusion, CBAM attention and gated selection: as modules were progressively introduced, overall performance metrics improved and the full model achieved the best results across multiple evaluation settings. Comparative experiments showed that MedSwinNet provided superior overall performance and stability relative to mainstream backbones including ResNet34, DenseNet, GoogLeNet and MaxViT. In addition, clinician readings were incorporated as a clinical baseline. Clinicians exhibited relatively high sensitivity but lower specificity and precision in the single‐kidney task (suggesting a tendency towards ‘positive’ calls), whereas in the bilateral‐kidney task they showed higher specificity and precision but reduced sensitivity (suggesting a more conservative strategy). A descriptive reference comparison indicated that MedSwinNet achieved higher composite metrics than the clinician baseline in both tasks, supporting its potential value for decision support. It should be emphasised, however, that model evaluation in this study was based on 10‐fold cross‐validation and the reported model results reflect fold‐aggregated averaged confusion matrices and reconstructed metrics rather than per‐case predictions strictly aligned with clinician readings; therefore, the ‘clinician vs. model’ comparison is descriptive and does not support rigorous paired statistical inference.

In summary, the main contributions of this work are as follows: (1) we developed a DMSA‐oriented VUR prediction framework and an enhanced network (MedSwinNet) that improves discrimination under weak‐signal conditions via multi‐scale fusion, attention‐based recalibration and gated selection; (2) we conducted a systematic evaluation on real‐world clinical data and reduced the risk of performance inflation due to image‐quality drift through QC consistency analyses; and (3) we provided representative difficult‐case comparisons to illustrate model robustness under challenging conditions such as high noise and low contrast. Collectively, this method may serve as a pre‐VCUG/MCU risk‐stratification tool to support reduction of unnecessary invasive examinations.

This study remains limited by its single‐centre retrospective design. Future work will pursue multicentre external validation and incorporate explainability visualisation together with calibration assessment and clinical net‐benefit evaluation (e.g., calibration curves and decision curve analysis) to further improve interpretability and clinical usability.

## Author Contributions

**Kai Zhao:** writing – review and editing, resources. **Zeyu Cui:** software, writing – original draft, formal analysis, visualization, validation. **Yunlong Zhou:** writing – review and editing, project administration, resources. **Yifan Luo:** data curation, investigation. **Dexuan Wang:** writing – review and editing, funding acquisition, conceptualization. **Zhantian Zhang:** data curation, investigation, validation. **Hongzhou Lin:** writing – original draft, data curation, formal analysis, investigation. **Jianwei Shuai:** methodology, funding acquisition, writing – review and editing, supervision, project administration, conceptualization.

## Funding

This work was supported by the National Natural Science Foundation of China (Grant U24A2014), the Natural Science Foundation of Zhejiang Province (Grant LY17H050006), and the Wenzhou Science and Technology Bureau Project (Grant ZY2024002).

## Conflicts of Interest

The authors declare no conflicts of interest.

## Data Availability

The source code is available online at https://github.com/cuizeyucuizeyu/MedSwinNet.

## References

[1] N. Shaikh , J. C. Craig , M. M. Rovers , et al., “Identification of Children and Adolescents at Risk for Renal Scarring After a First Urinary Tract Infection: A Meta‐Analysis With Individual Patient Data,” JAMA Pediatrics 168, no. 10 (2014): 893–900. 25089634 10.1001/jamapediatrics.2014.637

[2] A. M. Arlen , T. Leong , A. J. Kirsch , and C. S. Cooper , “Spontaneous Vesicoureteral Reflux Resolution Curves Based on Ureteral Diameter Ratio,” Journal of Pediatric Urology 19, no. 4 (2023): 468.e1–468.e6. 10.1016/j.jpurol.2023.04.028 37188603

[3] R. Stein , H. S. Dogan , P. Hoebeke , et al., “Urinary Tract Infections in Children: EAU/ESPU Guidelines,” European Urology 67, no. 3 (2015): 546–558. 25477258 10.1016/j.eururo.2014.11.007

[4] R. Chaudhry , P. P. Dangle , G. M. Cannon , F. X. Schneck , and H. A. Stephany , “Prospective Evaluation of Radiation Dose With Conventional Fluoroscopic Voiding Cystourethrogram in Pediatric Patients,” Journal of Pediatric Urology 17, no. 6 (2021): 790.e1–790.e5. 10.1016/j.jpurol.2021.09.010 34629302

[5] C. Rongviriyapanich and N. Pongnapang , “Radiation Doses to Paediatric Patients Undergoing Voiding Cystourethrography (VCUG) at Siriraj Hospital,” Siriraj Medical Journal 68, no. 2 (2016): 84–89.

[6] X. Feng , P. Puri , and M. Lacher , “Vesicoureteral Reflux (VUR),” in Pearls and Tricks in Pediatric Surgery (Springer International Publishing, 2021), 331–338.

[7] S. J. Jang , B. S. Choi , and S. H. Choi , “Evaluation of Renal Function in Obstructed Ureter Model Using 99mTc‐DMSA,” In Vivo 34, no. 5 (2020): 2431–2435. 32871769 10.21873/invivo.12057 PMC7652498

[8] D. Chroustová , J. Trnka , J. Langer , I. Urbanová , L. Lambert , and R. Kočvara , “How the 99mTc‐DMSA Scintigraphy Findings Are Reflected in the Adopted Treatment of Primary Vesicoureteral Reflux: One Centre Experience,” Journal of Pediatric Urology 19, no. 4 (2023): 456–462. 36870928 10.1016/j.jpurol.2023.01.017

[9] D. Plyku , M. Ghaly , Y. Li , et al., “Renal 99mTc‐DMSA Pharmacokinetics in Pediatric Patients,” EJNMMI Physics 8, no. 1 (2021): 53. 34283316 10.1186/s40658-021-00401-7 PMC8292521

[10] T. Smith , K. Evans , M. F. Lythgoe , P. J. Anderson , and I. Gordon , “Radiation Dosimetry of Technetium‐99m‐DMSA in Children,” Journal of Nuclear Medicine 37, no. 8 (1996): 1336–1342. 8708767

[11] R. Wang , F. Chen , H. Chen , et al., “Deep Learning in Digital Breast Tomosynthesis: Current Status, Challenges and Future Trends,” MedComm 6, no. 6 (2025): e70247. 40491967 10.1002/mco2.70247 PMC12146671

[12] Y. Tang , D. Yang , W. Li , et al., “Self‐Supervised Pre‐Training of Swin Transformers for 3D Medical Image Analysis,” in IEEE/CVF Conference on Computer Vision and Pattern Recognition (CVPR) (2022), 20740–20750.

[13] W. Chen , S. Ren , K. Guo , et al., “High‐Resolution Swin Transformer for Automatic Medical Image Segmentation,” Sensors 23, no. 7 (2023): 3420. 37050479 10.3390/s23073420 PMC10099222

[14] L. Zhang , X. Yin , X. Liu , and Z. Liu , “Medical Image Segmentation by Combining Feature Enhancement Swin Transformer and UperNet,” Scientific Reports 15 (2025): 14565. 40281077 10.1038/s41598-025-97779-6 PMC12032031

[15] A. Masood , U. Naseem , and J. Kim , “Multi‐Level Swin Transformer Enabled Automatic Segmentation and Classification of Breast Metastases,” in IEEE Engineering in Medicine and Biology Society (EMBC) (IEEE, 2023), 1–4. 10.1109/EMBC40787.2023.10340831 38082574

[16] O. Ergün , T. A. Serel , S. A. Öztürk , H. B. Serel , S. Soyupek , and B. Hoşcan , “Deep‐Learning‐Based Diagnosis and Grading of Vesicoureteral Reflux: A Novel Approach for Improved Clinical Decision‐Making,” Journal of Surgery and Medicine 8, no. 1 (2024): 12–16.

[17] Z. Li , Z. Tan , Z. Wang , et al., “Development and Multi‐Institutional Validation of a Deep Learning Model for Grading of Vesicoureteral Reflux on Voiding Cystourethrogram: A Retrospective Multicenter Study,” EClinicalMedicine 69 (2024): 102466. 38361995 10.1016/j.eclinm.2024.102466 PMC10867607

[18] Y. Eroglu , K. Yildirim , A. Cinar , and M. Yildirim , “Diagnosis and Grading of Vesicoureteral Reflux on Voiding Cystourethrography Images in Children Using a Deep Hybrid Model,” Computer Methods and Programs in Biomedicine 210 (2021): 106369. 34474195 10.1016/j.cmpb.2021.106369

[19] G. Chen , L. Su , S. Wang , et al., “Automated Grading of Vesicoureteral Reflux (VUR) Using a Dual‐Stream CNN Model With Deep Supervision,” Journal of Imaging Informatics in Medicine 38, no. 6 (2025): 3517–3525. 39953261 10.1007/s10278-025-01438-1 PMC12701105

[20] S. Kabir , J. L. Pippi Salle , M. E. H. Chowdhury , and T. O. Abbas , “Quantification of Vesicoureteral Reflux Using Machine Learning,” Journal of Pediatric Urology 20, no. 2 (2024): 257–264. 37980211 10.1016/j.jpurol.2023.10.030

[21] S. Woo , J. Park , J. Y. Lee , and I. S. Kweon , “CBAM: Convolutional Block Attention Module,” in European Conference on Computer Vision (Springer International Publishing, 2018), 3–19.

[22] W. Wu , X. Yang , C. Yao , O. Liu , Q. Zhao , and J. Shuai , “E‐SegNet: E‐Shaped Structure Networks for Accurate 2D and 3D Medical Image Segmentation,” Research 8 (2025): 869. 10.34133/research.0869 PMC12408157 40909238

[23] I. Loshchilov and F. Hutter , “Decoupled Weight Decay Regularization,” in International Conference on Learning Representations (2019).

[24] D. P. Kingma and J. Ba , “Adam: A Method for Stochastic Optimization,” in International Conference on Learning Representations (2015).

[25] L. Prechelt , “Early Stopping—But When?,” in Neural Networks: Tricks of the Trade (Springer Berlin Heidelberg, 1998), 55–69.

[26] L. Prechelt , “Early Stopping—But When?,” in Neural Networks: Tricks of the Trade: Reloaded (Springer Berlin Heidelberg, 2012), 53–67.

[27] I. Goodfellow , Y. Bengio , and A. Courville , Deep Learning (MIT Press, 2016).

[28] C. M. Bishop , Pattern Recognition and Machine Learning (Springer, 2006).

[29] K. P. Murphy , Machine Learning: A Probabilistic Perspective (MIT Press, 2012).

[30] K. He , X. Zhang , S. Ren , et al., “Deep Residual Learning for Image Recognition,” in IEEE Conference on Computer Vision and Pattern Recognition (CVPR) (2016), 770–778.

[31] G. Huang , Z. Liu , L. van der Maaten , et al., “Densely Connected Convolutional Networks,” in IEEE Conference on Computer Vision and Pattern Recognition (CVPR) (2017), 4700–4708.

[32] C. Szegedy , W. Liu , Y. Jia , et al., “Going Deeper With Convolutions,” in IEEE Conference on Computer Vision and Pattern Recognition (CVPR) (2015), 1–9.

[33] Z. Tu , H. Talebi , H. Zhang , et al., “MaxViT: Multi‐Axis Vision Transformer, Computer Vision,” in European Conference on Computer Vision (Springer Nature Switzerland, 2022), 459–479.

[34] P. Xu , J. H. Chen , Y. K. Fu , et al., “Electronic Skin Pressure Map Mask Generation Based on Optimised Real‐ESRGAN‐MSDC,” Chinese Physics B (2026); in press.

[35] G. S. Collins , P. Dhiman , C. L. A. Navarro , et al., “TRIPOD+AI Statement: Updated Guidance for Reporting Clinical Prediction Model Studies That Use Regression or Machine Learning Methods,” BMJ 385 (2024): e078378. 38626948 10.1136/bmj-2023-078378 PMC11019967

[36] B. Van Calster , D. J. McLernon , M. van Smeden , et al., “Calibration: The Achilles Heel of Predictive Analytics,” BMC Medicine 17, no. 1 (2019): 230. 31842878 10.1186/s12916-019-1466-7 PMC6912996

[37] A. J. Vickers and E. B. Elkin , “Decision Curve Analysis: A Novel Method for Evaluating Prediction Models,” Medical Decision Making 26, no. 6 (2006): 565–574. 17099194 10.1177/0272989X06295361 PMC2577036

[38] T. Han , Z. Pan , W. Ge , and Q. Zhao , “Multi‐Granularity Fusion Model With Mamba Architecture for Ames Mutagenicity Prediction,” Journal of Medicinal Chemistry 69, no. 2 (2026): 1766–1778. 41489521 10.1021/acs.jmedchem.5c03551

[39] Z. Pan , X. Yang , C. Wang , T. Han , and Q. Zhao , “Multimodal Feature Fusion for Bone Toxicity Prediction and Local Platform,” Journal of Chemical Information and Modeling 65, no. 23 (2025): 12799–12810. 41264781 10.1021/acs.jcim.5c02280

[40] R. Liu , Z. Wang , T. Han , L. Shao , and Q. Zhao , “HetGAT‐LMI: Interpretable Heterogeneous Graph Attention Method for Predicting lncRNA‐miRNA Interactions,” Journal of Chemical Information and Modeling 66, no. 1 (2026): 796–807. 41432189 10.1021/acs.jcim.5c02664

[41] F. Zhu , Q. Niu , X. Li , Q. Zhao , H. Su , and J. Shuai , “FM‐FCN: A Neural Network With Filtering Modules for Accurate Vital Signs Extraction,” Research 7 (2024): 361. 10.34133/research.0361 PMC11082448 38737196

[42] Q. He , X. Li , J. Zhong , G. Yang , J. Han , and J. Shuai , “Dear‐PSM: A Deep Learning‐Based Peptide Search Engine Enables Full Database Search for Proteomics,” Smart Medicine 3, no. 3 (2024): e20240014. 39420951 10.1002/SMMD.20240014 PMC11425048

[43] Q. C. Li , H. Lin , P. Wang , et al., “Analysis of Spatiotemporal Dynamic Patterns of Gene Expression During Mouse Embryonic Development Based on Moran's *I* and Spatial Transcriptomics,” Chinese Physics B 34 (2025): 088703.

[44] F. Xu , X. Li , R. Wu , et al., “Incoherent Feedforward Loop Dominates the Robustness and Tunability of Necroptosis Biphasic, Emergent, and Coexistent Dynamics,” Fundamental Research (2024); in press. 10.1016/j.fmre.2024.02.009 PMC13069638 41971832

